# Supplementary material for: Are carbon emissions trading and green financial instruments synergistic? -Comprehensive quantitative research based on content analysis
Source: PLoS One. 2024 Mar 7;19(3):e0298601. doi: 10.1371/journal.pone.0298601 (PMC10919601; doi:10.1371/journal.pone.0298601)
Supplement: S1 Data — (ZIP) [file pone.0298601.s001.zip › Data/地级市Z_F工作报告.zip!/╣π╢1⁄2/╒╪╟∞╩╨/└┤╫╘╣1⁄2╓┌║┼í╛┬φ┐╦╩2╛▌═°í┐_2008.docx]

各位代表：

我代表肇庆市人民政府向大会作政府工作报告，请予审议，并请政协委员和其他列席人员提出意见。

2007年工作回顾

去年是本届政府的开局之年。一年来，在中共广东省委、省人民政府和中共肇庆市委的正确领导下，在市人大及其常委会和市政协的监督支持下，市政府坚持以邓小平理论和“三个代表”重要思想为指导，以科学发展观统领经济社会发展全局，认真贯彻落实党的十七大精神，按照市第十次党代会的战略部署，团结带领全市人民解放思想，抢抓机遇，乘势而上，扎实工作，大力实施“五大发展战略”，加速发展“四大经济”，积极落实“五大民生工程”，推动经济社会又好又快发展，全面完成或超额完成了市十一届人大一次会议确定的各项目标任务。

——经济发展再创新高。本地生产总值实现616.55亿元（快报数，下同），完成预期目标的101.1%，比上年增长15.3%，创近十年最高增幅，比全省平均增幅高出0.8个百分点。

——工业经济提速增效。规模以上工业增加值实现157.9亿元，比上年增长34.5%，增幅在全省排名第四；实现工业利润和税金分别增长29%和45%。

——县域经济实力增强。6个县（市）有4个规模以上工业增加值增幅超过30%，县域地方财政一般预算收入合计17.76亿元，比上年增长28.9%，创近年最好水平。全市农业总产值实现247.27亿元，增长5.6%。高要、四会综合增长率在全省列入考核的67个县（市）中分别排第一、第六名。

——第三产业发展加快。全市主要景区接待游客1152万人次，旅游总收入55.3亿元，分别比上年增长12.9%和15.4%。房地产开发投资和商品房销售额分别增长84.3%和72.7%。外贸出口总额21.73亿美元，增长23.3%。社会消费品零售总额196.83亿元，完成预期目标的101.9%，增长17.2%。第三产业增加值实现251.71亿元，增长14%。

——重大项目建设有新突破。纳入重点跟踪落实的35个重大项目完成投资89亿元。二广高速公路三水至怀集段全线施工，肇庆新港开港营运，首家五星级标准酒店建成试业。全社会固定资产投资完成293.42亿元，完成预期目标的112.8%，比上年增长35.3%。

——招商引资上新台阶。新批外商投资项目312宗，合同外资额13.69亿美元，比上年增长23.3%；实际吸收外资7.75亿美元，完成预期目标的102.1%，增长15.3%，总量居全省东西两翼和山区13个市首位。

——城乡建设迈出新步。城市规划建设管理水平得到提升，肇庆城东新区基础设施建设一期工程等一批重大市政项目基本完成。社会主义新农村建设加快推进，创建生态文明村580个，新建省卫生村278个。

——节能减排成效明显。关停淘汰落后生产能力企业32家，削减二氧化硫1500吨、化学需氧量1750吨。规模以上工业增加值能耗下降9%，万元GDP能耗下降4%，超过年均下降3.43%的目标。

——财税收入大幅增长。地方财政一般预算收入实现32.74亿元，完成预期目标的114%，比上年增长31.1%，增幅在全省排名第七，创实施分税制以来最好水平。国税、地税收入分别增长30%和34.9%。

——人民生活不断提高。城镇居民人均可支配收入12794元，比上年增长10.8%；农村居民人均纯收入5010元，增长5%。年末城乡居民储蓄存款余额比年初增长8%。全年居民消费价格总指数上升2.7个百分点，涨幅低于全国、全省平均水平。

在实现经济又好又快发展的同时，社会各项事业全面发展，社会和谐稳定。一年来，市政府和各级、各部门坚持“继承、创新、发展”的工作方针，增强了工作的紧迫感和责任感，团结拼搏、加油鼓劲、开拓进取、攻坚破难，促进各项工作落实。

一、全面发展“四大经济”，着力提高经济发展水平

重点发展工业经济。深入实施“工业主导战略”，制定实施加快工业发展的考核奖励办法，推动产业优化升级，促进产业集群化和适度重型化，电子信息、生物制药、食品饮料、金属加工、汽车配件、林产化工六大支柱产业实现总产值占规模以上工业的50%，重工业实现总产值占规模以上工业的67%。积极引进和推动工业项目建设，规模以上工业企业增加118家，总数达906家。加强企业技术进步，在建工业技改项目115个，总投资22.75亿元，增资扩产项目60项，总投资20.66亿元。积极实施名牌带动战略和推动企业上市，风华公司磁性材料成为我市第一个中国名牌产品，国家免检产品已有7个，省级工业类名牌产品和著名商标分别增加到37个、22件，确定了30家上市后备企业。中心板块和东南板块工业发展势头迅猛，肇庆高新区新投产企业50家，亚洲铝业成为我市首家年产值超50亿元的企业，高要小五金、精密压铸、汽配产业基地，四会省级民营科技园，端州三榕工业园和鼎湖纺织鞋业基地等加快建设，高要、四会、肇庆高新区工业总产值均超过100亿元。企业规模效益不断壮大，全市年税收超千万元工业企业达到34家，规模以上工业经济效益综合指数提高23个百分点。

加快发展城市经济。积极推进旅游资源整合开发和旅游配套设施建设，成功举办“2007广东国际旅游文化节·肇庆嘉年华”等活动，荣获“中国最佳休闲旅游城市”、“中国最受欢迎旅游目的地”、“中国旅游文化示范地”称号，“南国旅游休闲之都”旅游品牌形象和知名度不断提高。促进房地产业健康发展，端州、鼎湖房地产企业上缴税收分别比上年增长36.6%和73%。狠抓产品质量和食品安全专项整治，实现了12个100%目标。落实“打假”工作责任制，改善消费环境，促进了商贸流通服务业发展。

发展壮大县域经济。实行以资源引资金、引技术，一批新上项目和技改扩产项目动工建设或投产，推动县域工业提速发展。德庆林产化工加工基地规模效益迈上新水平，封开新引进华润年产1200万吨水泥项目，广宁林浆纸一体化产业集聚发展，怀集钢铁、山梨糖醇等资源型工业不断壮大，德庆、封开和怀集初步形成人造板加工产业集群。4个山区县新投产企业增加工业产值约20亿元。落实各项支农惠农政策，共争取国家和省财政扶持农、林、水各业资金3.7亿元，市、县两级财政投入农、林、水各业资金1.84亿元。全面落实种粮补贴政策，完成了省下达的粮食考评任务。优化农业结构，扩种柑桔12万亩，全省柑桔第一大市地位得到巩固，德庆、封开分别获“中国柑桔之乡”、“中国油栗之乡”称号，四会沙糖桔和贡柑成功注册原产地证明商标。做好动植物重大疫病防控工作，畜牧、优质蔬菜和水产养殖业规模扩大，四会成为全省生猪生产第一大县（市），高要被授予“中国罗非鱼之乡”、“中国罗氏虾之乡”，德庆获“中国果菜无公害科技示范基地”称号。加快农业产业化，新建成省级重点农业龙头企业3户、市级农业龙头企业12户和无公害农业生产基地11个，新增省级农业名牌产品3个。

培育发展港口物流经济。制定实施加快发展港口物流经济的政策措施，召开了高规格的港口物流经济发展座谈会，港口基础设施和临港产业园建设抓紧推进，完成了肇庆新港首期工程以及进港公路建设，肇庆新港物流基地已开展规划和征地，大旺珠江物流园启动建设，肇庆港口货物吞吐量1364.2万吨，比上年增长130%，通过肇庆口岸进出口货物总值增长32.7%。

二、大力招商引资，着力发展园区经济

继续把招商引资作为加快发展的主引擎，积极创新方式、拓宽领域、优化结构，掀起招商引资新高潮。组织参加赴中东、非洲、澳大利亚、新西兰、马来西亚、日本、韩国以及广州、佛山等经贸活动和2007粤港、粤台经贸交流会、广东国际旅游文化节泛珠三角旅游招商会。2007“肇庆金秋”经贸洽谈会取得圆满成功，签约、奠基和投产项目共169宗，投资总额425.36亿元，引进投资50亿元的中国国电集团风能设备制造及热电联产、投资15亿元的四会志高家电研发等一批大项目。加快招商引资载体建设，促进园区经济快速发展。肇庆高新区新签项目61宗，投资总额19.8亿美元，其中千万美元以上的大项目突破50宗，实际吸收外资突破3亿美元，连续三年在全省省级开发区中名列第一，本地生产总值、规模以上工业增加值、财政一般预算收入分别增长82.7%、100.1%、98.4%。市区共建的鼎湖高新产业集聚基地已完成控制性详细规划。产业转移园区建设取得明显成效，顺德龙江（德庆）工业园企业17家，中山大涌（怀集）工业园签约企业14家，佛山大沥（广宁）、广州黄埔（封开）和佛山禅城（高要）产业转移工业园正在申报。认真落实加快中小企业和民营经济发展的政策措施，积极组织企业参加中博会等经贸交流活动，推动民营经济加快发展，全年规模以上民营工业总产值263.66亿元，个体私营企业上缴税金10.5亿元，分别比上年增长36%和33.8%.

三、积极谋划建设重大项目，着力增强区域竞争力

积极实施“重大项目带动战略”，亚洲金属资源再生工业基地通过国家环保总局验收，签约企业40家，已建成12家；高新区亚洲铝业二期、德庆石井水泥、高要新明珠建材等重大工业项目建设进展顺利。广宁20万吨燃料乙醇项目列入省“十一五”专项规划，鼎丰纸业林浆纸一体化项目完成环境评价报告。二广高速公路三水到怀集段完成总投资额的四分之一。国道321线高要小湘至封开涌口段文明样板路全面竣工，三水至四会马房段改造工程正式动工。珠外环高速公路肇庆段前期工作基本完成。南广铁路、贵广铁路经我市的线路规划走向已基本确定。西江航道整治肇庆至封开界首段已完成前期工作。景丰联围加固工程继续推进，大旺围北江段加固工程基本完成。肇庆燃煤电厂列入省今年重点能源建设项目。肇庆核电项目进入全面可研阶段。肇庆抽水蓄能电站列为南方电网公司候选站址。

四、加强城乡规划建设和生态环保，着力优化发展和人居环境

编制完成了《肇庆市城市总体规划修编（2006－2025）》和《肇庆市中心区域协调发展规划》纲要，批准实施《肇庆市龟顶新城区控制性详细规划》、《肇庆市端州城区中小学布点规划》。城东新区城市设计、鼎湖区发展概念性规划和多个专项规划基本完成。加强城市建设和管理，加快推进中心城区东扩，肇庆城东新区基础设施建设二期工程启动，端州七、八路和星荷路改造等市政工程基本完成。在肇庆城区收储土地1700多亩，增强了城市经营能力。累计编制完成387个村庄的整治规划。在全省率先完成市级土地利用总体规划大纲框架编制工作。加强土地征用监管，制定实施肇庆城区和高新区闲置土地处理办法，全市收回土地使用权23宗，面积共1586亩。加大环保执法力度，严格环保准入，加强对重点污染源的监管，完成了星湖治污和鼎湖广利涌整治工程，启动四会独水河综合整治工作，90%以上企业实现达标排放。四会二期、高新区首期污水处理厂已动工建设。加强森林资源培育、保护和自然保护区建设，全市森林覆盖率提高到65.1%，城市人均公共绿地面积增加到9.5平方米，四会和高要分别被国家、省授予“绿色小康县（市）”和“林业生态县（市）”称号。

五、积极创新体制机制，着力破解经济发展瓶颈

加快建设公共财政管理体制，新增市级国库集中支付实施单位128家，开通实时在线财政预算监督系统，初步建立起贯穿预算编制、执行、监督全过程、覆盖预算内外全领域的公共财政支出管理体系。探索实行政府性投资项目“代建制”。推进了公共资源的企业化、市场化运作。加强国有资产监督营运，完善了国有企业预算管理和业绩考核机制。深化国有企业改革，市属101家国有企业实施了转制改革，妥善安置1979名职工。积极推进金融服务创新，加快城市信用社退市，深化农村信用社改革，组建中国邮储银行肇庆分行，金融产业发展壮大，中小企业信用担保机构增加到7家，累计为175家企业提供融资担保5.64亿元，年末金融机构人民币贷款余额比年初增长21.1%。完善鼎湖区管理体制，撤销大冲收费站，解决了多年制约肇庆城区经济发展的突出问题，促进了市区一体化建设。积极争取建设用地指标，坚持节约有效利用土地，为经济发展提供用地保障。积极应对电力供应紧张，通过采取科学调度、政府补贴促本地企业发电和实行错峰等办法，解决生产生活用电问题。完成封开、怀集、德庆三个代管县供电企业体制改革，实现全市各类用电同网同价。

六、扎实推进“五大民生工程”，着力解决群众生产生活问题

市、县两级投入“五大民生工程”财政性资金2.16亿元。抓好职业技能培训工程，实施各类技能培训58214人次，其中下岗失业人员再就业培训3712人，农村青年技能培训34517人。新增就业岗位37216个，农村转移就业达56406人，年末城镇登记失业率为2.54%。抓好教育均衡发展工程，多渠道投入资金5.4亿元，新增义务教育学位7.88万个、高中阶段学位1.5万个，全面完成了793所农村义务教育学校40多万平方米C、D级危房改造。端州区新建扩建学校4所，新增优质学位3800个。全面落实农村义务教育、城镇低保家庭义务教育免收学杂费和课本费政策，受惠学生58.6万人。抓好便民廉医工程，提高新型农村合作医疗保障水平，新农合覆盖率达84.23%，住院报销封顶线提高到1万元以上。加强乡镇卫生院和村卫生站建设，全面完成“一村一站”建设目标，新建规范化村卫生站178个，建成社区卫生服务站3个。抓好全民安居工程，全面完成1.1万农村贫困户危房改造，使5.5万人住上安全、实用的住房。着力解决端州城区困难家庭住房难问题，安排安居房903套。2932户业主旷时10多年的房产办证难问题得到基本解决。抓好生产生活环境改造工程，全面完成镇通行政村公路硬底化改造任务，累计实施3693公里，农村客运班线通达率90.1%、300万人受惠。完成18宗饮水安全工程设计并落实了建设资金。加大社会保障工作力度，社会保险费征缴率达97.54%，企业退休人员人均月基本养老金增加120元，提高到782元。人均年收入在1200元以下的困难群众全部纳入低保救济范围，实现了动态管理下的应保尽保，并因物价变化增发了临时性价格补贴。启动被征地农村养老保险工作，开展了城镇居民基本医疗保险试点。

七、全面发展社会各项事业，着力统筹经济社会协调发展

大力发展科技事业。加强科技创新平台建设，新建省级工程技术研发中心1家、省级企业技术中心2家，新认定高新技术企业32家、高新技术产品8个，通过专利申请358件、专利授权268件。成功举办产学研技术合作交流洽谈会，承办泛珠三角区域科技合作座谈会暨科技信息服务现场会，被列为广东省与教育部产学研结合项目10个、示范基地3个。创建2个省级技术创新专业镇。推进信息化工作，“农村信息直通车工程”应用推广走在全国前列。肇庆市、高要市分别通过国家科技进步考核并被评为全国市级和县级科技进步先进单位。

加快发展教育事业。不断提高“普九”水平，小学、初中适龄人口入学率分别为99.96%、98.27%。狠抓普通高中扩建扩招，普通高中招生人数增长9.57%、98.27%。狠抓普通高中扩建扩招，普通高中招生人数增长9.57%，4所学校通过国家级示范性高中考核验收，高考上线人数增幅超全省平均水平。大力发展职业技术教育，中等职业教育招生人数增长14.4%，在校生规模全省排名第三，民办职业教育在校生人数和校均规模均居全省首位。加快发展高等教育，普通高校（含高职）招生人数增长23%。

繁荣发展文化事业。实施文化信息资源共享、农村电影放映、广播电视村村通和农家书屋建设“四大文化工程”。建成市、县、镇、村四级公共文化服务网络。完成296个20户以上已通电自然村通广播电视工作。文艺精品不断涌现，四会创作的《桔花缘》获全国“五个一工程”奖。宋城墙修缮工程基本完成。第一批19个项目被确认为市级非物质文化遗产保护名录，8个项目被列入第二批省级非物质文化遗产代表作。

积极发展各项社会事业。加强公共卫生和基本医疗服务体系建设，疾病预防控制机构建设项目即将完成，卫生应急预案框架基本建立，城乡卫生服务水平提高，市中医院创建三级甲等中医院通过省级评审。全民健身活动广泛开展，竞技体育得到加强，成功举办市第八届运动会和肇庆国际龙舟邀请赛，承办多项全国重大体育赛事。全面完成了省下达的人口计划。社会中介和民间组织管理工作继续加强。创新慈善工作机制，组织系列公益慈善活动40多场，筹得款物3480万元。民族、宗教、外事、侨务、国防动员、人防、妇女儿童、老龄、残疾人、档案等工作取得新成绩。

努力维护社会稳定。抓好“五五”普法，推进依法治市，增强全民法制意识。落实维护第一责任，开展21项民生信访问题专项治理，及时排查调处社会矛盾纠纷，积极预防和妥善处置群体性事件。强化安全生产责任制，安全生产形势平稳。加强社会治安综合治理，依法打击各类犯罪活动，群众安全感进一步提高，再次被评为省社会治安综合治理优秀市。

八、切实加强政府自身建设，着力提升公共服务效能

认真执行市人大及其常委会的决议，向市人大及其常委会提交各类专项工作报告13件，组织办理人大代表建议83件。向市政协通报情况，组织办理政协提案144件。审计、法制工作不断加强。办结行政复议申请38件。强化了政府公共服务和应急处置能力。组织了肇庆城区污水处理费价格调整、大冲收费站撤并等重大事项社会公开听证。建立电子政务和电子监察系统，310个单位全部实施政务公开，市行政服务中心公开了市直48个部门、497个审批事项申请指引，行政审批事项按时办结率达99%。在全国率先创建“农村党风廉政建设信息公开平台”，打造“阳光村务”。开通全省第一条政务公开电话热线，为群众提供51个单位、292条办事流程和370条审批流程的查询服务。深入开展民主评议政风和机关作风建设测评活动，“行风热线”栏目受理解决投诉1440宗。中央、省驻肇单位为地方经济社会发展作出了新贡献。

各位代表，去年政府工作目标任务的顺利完成，是省委、省政府、市委正确领导和市人大、市政协监督支持的结果，同时凝聚着全市上下、社会各方的智慧和汗水。在此，我代表市人民政府，向全市工人、农民、干部、知识分子、外来务工人员、驻肇单位、驻肇部队、武警官兵以及各界人士致以崇高的敬意！

在充分肯定成绩的同时，我们也清醒地看到存在的困难和和问题：经济总量仍然不大，产业结构不够优化，发展质量有待提高；区域发展不平衡，山区县域经济基础薄弱，农民增收缓慢，农村公共事业建设滞后；制约经济发展因素增多，立项报批难、土地和电力供应紧张、资金筹措难、企业招工难、生产经营成本持续上升等问题较突出；民生问题仍然较多，居民收入渠道较窄，社会保障水平还不高，部分低收入群众生活比较困难；政府职能转变还没有完全到位，公共服务和社会管理水平还不高，机关作风建设有待进一步加强。我们必须高度重视，采取有力措施加以解决。

2008年工作安排

今年是我市全面贯彻党的十七大精神，落实省、市第十次党代会决策部署，实现科学发展、构建和谐社会的重要一年。我们必须把握形势，扬长避短，调整思路，科学谋划。当前，经济全球化和区域经济一体化趋势更加明显，我国国际地位的提升，我省区域协调发展战略实施力度加大等都为我市招商引资和扩大承接珠三角等发达地区产业转移、加快融入广佛经济圈提供了难得的机遇。特别是在大珠三角、泛珠三角区域合作发展进程中，我市的区位、资源、交通、生态优势更加突出，加快发展具备了良好的基础和条件。但是，我们也要看到，区域竞争在加剧，我市经济基础薄弱、历史欠账多的问题仍未有效解决，制约发展的困难和问题较突出，社会建设和改善民生的任务仍很艰巨。我们要认真贯彻落实市委十届四次全会精神，紧紧把握争当实践科学发展观排头兵的新要求，以新一轮思想大解放推动新一轮大发展。破除“小富则安”思想，增强进取意识；破除“小进则骄”思想，增强忧患意识；破除“因循守旧”思想，增强创新意识；破除“畏难却步”思想，增强拼搏意识；破除“各自为政”思想，增强全局意识。坚定发展信心，积极抢抓机遇，主动应对挑战，着力破解难题，正确处理好经济发展中“好”与“快”的关系，经济与社会发展的关系，统筹区域城乡协调发展的关系，加快发展与环境保护的关系。加强学习，转变作风，真抓实干，以更饱满的热情、更紧迫的责任感和更强有力的措施做好工作，推动经济社会全面转入科学发展轨道，努力走在全省实践科学发展观前列，为广东争当实践科学发展观排头兵作出肇庆应有贡献。

今年政府工作的指导思想是：全面贯彻党的十七大精神，深入落实科学发展观，按照省委十届二次全会、市第十次党代会和市委十届四次全会的部署，继续解放思想，坚持改革开放，以经济建设为中心，狠抓发展第一要务，转变发展方式，全面推进“五大发展战略”，加速发展“四大经济”，深入实施“五大民生工程”，加强社会事业建设，着力构建和谐社会，实现经济社会又好又快发展，加快建设繁荣活力、文明法治、和谐安康、生态环保肇庆，为全面建设小康社会打下坚实基础。

今年经济社会发展主要预期目标是：本地生产总值增长14%，一、二、三产业增加值分别增长5%、22.2%、13.5%，工业增加值增长24.2%，全社会固定资产投资增长18%，地方财政一般预算收入增长15%，社会消费品零售总额增长15%，外贸出口增长12%，实际吸收外资增长10%，万元GDP能耗降低3.45%，城镇登记失业率控制在3%以下，人口自然增长率控制在省下达指标以内，城镇居民人均可支配收入增长9%，农村居民人均纯收入增长6%，居民消费价格总指数涨幅控制在4%以内。

一、狠抓发展第一要务，推动经济又好又快发展

（一）积极推动产业集群发展和优化升级，做大做强工业经济。加强产业集群发展规划，重点发展电子信息、金属加工、机械和汽车配件三大主导产业，生物工程、造纸和木材加工、食品饮料三大优势产业和新型建材、林产化工、纺织三大传统产业，争取建立省市共建的汽车配套产业制造基地。落实100户重点企业跟踪服务制度，完善激励机制，促进风华高科、蓝带啤酒等规模企业发挥生产能力，支持亚洲铝业、鼎丰纸业、飘雪鼎湖山泉等重点企业扩产发展，推动天龙油墨、同步化工、鸿特压铸、华锋电子等优强企业上市融资，努力打造一批年产值超10亿元、50亿元、100亿元，创税超亿元、5亿元、10亿元的工业龙头企业。优化产业结构，发展新型工业，推动企业自主创新，组织一批对产业产品结构升级起关键作用的技改项目，突出抓好投资500万元以上重点技改项目，争取建立肇庆动力配件、广东鸿图2个国家级企业技术中心和一批省级企业技术中心，推动企业加强技术开发和成果转化，提高产品的科技含量和附加值。促进企业加强管理，提高经济效益。加大名牌创建力度，再创一批国家级、省级名牌产品、驰（著）名商标。加大重点工业园区资源整合和开发建设力度，以肇庆高新区为重点促进园区经济加快发展。

（二）努力提高招商引资水平，发展壮大内外源经济。坚持“走出去、请进来”策略，继续组织和参加境内外的各项招商推介活动，积极承接珠三角产业转移，力争招商引资取得新突破。建立不同区域招商引资评价机制和准入标准，优化招商引资结构，中心板块和东南板块要适当提高项目准入门槛，积极引进科技型、创税型、生态型项目；山区板块要发挥资源优势，大力引进资源型、创税型、生态型项目。拓展招商引资领域，加大旅游、物流、金融等现代服务业招商力度。引导外资、民资企业增资扩产和技术改造。加强项目跟踪落实，促进如期竣工投产和达标达产。加强进出口贸易，深化“大通关”口岸建设，重点扶持知名品牌和附加值高的产品出口，支持扩大进口，推动外贸发展由出口导向型向进出口均衡型转变。引导支持民营企业创立品牌、创新技术、创新体制，拓宽民营企业融资渠道，推动民营经济发展上新台阶。

（三）加速发展县域经济，加快建设社会主义新农村。落实各项强农惠农政策，发展现代农业，促进农业增效、农民增收。加强农业基础设施建设，抓好农田水利建设议案项目和农业综合开发土地治理项目实施，改造中低产田4.5万亩，启动1个市级、完成2个县级标准农田示范区建设。确保完成粮食考评任务。扶持农业龙头企业，争取肇庆柑桔产业列入国家产业带，加快禽畜水产养殖基地建设，争创2—3个省级农业名牌。强化动植物重大疫病防控和农产品质量安全监管，建设鼎湖沙浦、高要蚬岗等农产品无公害生产基地，争取50%农产品通过无公害认证。稳妥推进集体林权制度改革及各项配套改革，积极发展林业，扩大珍贵用材林、城市绿化苗木、速生丰产林三大基地，建立林权交易服务中心和珍贵树种产品交易专业市场。做大做强县域工业，力争所有山区县都与发达地区共建产业转移园区，做好二广高速公路、珠外环高速公路沿线产业集聚基地规划建设，开发封开铜钼矿等资源型产业，适度发展劳动密集型产业。加强县域和中心镇建设，繁荣发展县域特色旅游、商贸物流、房地产等第三产业。继续抓好专业镇建设规划，再造一批产业强镇。组织实施绿色富民示范工程。深入开展生态文明村和省卫生村创建活动，力争分别新建500个和300个。

（四）培育发展现代服务业，促进“三产旺市”。提升旅游业发展水平，深化星湖景区经营管理体制改革，启动星湖东门广场和肇庆城区旅游购物步行街规划建设，抓好千里旅游画廊新景区景点开发，培育“星湖夜游、中国砚都游、森林度假游、生态科普游”等特色旅游，拓展“文化街、文化广场和古村落游”等文化旅游。积极发展港口物流业，做好港口岸线规划、物流发展战略规划，推进肇庆新港综合物流基地立项、报批和土地调整工作，力争有一批物流企业进入全省2A以上等级物流企业行动。落实扩大内需政策，扶持发展商贸龙头企业，推动中高档酒店建设，扩大汽车销售专业市场辐射面。实施“万村千乡市场工程”和“双百市场工程”，促进城乡扩大消费。加强房地产业规划管理和政策引导，优化房地产结构，提高住宅小区开发建设水平，促进房地产业持续健康发展。加强政银企合作平台建设，争取股份制商业银行到我市设立分支机构，继续组建和完善信用担保机构，促进金融产业发展。推进社会信用体系建设，妥善处置城市信用社退市，切实防范金融风险。

（五）加强重大项目和城市规划建设管理，进一步增强发展后劲。继续谋划和加快重大项目建设，做好广宁鼎丰纸业扩建和燃料乙醇、封开华润水泥等项目的立项申报。促进亚洲金属资源再生工业基地二期、德庆石井水泥二期、国电风能设备制造和热电联产、碧桂园高档家具总部、四会志高家电研发、高要浙江百汇高分子材料等重大工业项目动工建设。推动二广高速公路三水至怀集段工程。配合实施珠外环高速江门至肇庆段、广肇高速二期、广梧高速封开连接线工程。推进国道321线四会马房至高要小湘段文明样板路改造和省道263线四会至广西交界段文明样板路创建。支持配合贵广铁路和南广铁路肇庆段规划建设。协调加快西江航道肇庆至封开界首段综合整治工程。推进肇庆新港配备设施建设。力争肇庆燃煤电厂通过国家核准并动工建设。做好核电、抽水蓄能电站前期工作。推进景丰联围、高要联金大堤等重点水利工程建设。加强城市规划建设管理，认真编制肇庆城东新区和鼎湖区控制性详细规划，推动端州城区交通、市政、管网等公共设施向鼎湖延伸，促进市区一体化。加快肇庆城东新区基础设施建设二期工程和人民北路、星湖大道、信安大道以及大冲广场等市政建设改造工程，启动龟顶新城等项目建设，推进肇庆城区管道燃气项目。组建肇庆城市管理综合执法队伍，提高城市综合管理水平。

（六）加强沟通协调，着力解决制约发展的瓶颈问题。加强部门之间、上下级之间的沟通协调，努力化解矛盾、破解难题。加快土地利用总体规划修编，积极向省争取用地规模和指标。科学合理配置建设项目和土地资源，严格控制项目用地规模，集约节约用地，提高单位土地的投资强度和对财政贡献率，依法清理闲置土地和低效用地，千方百计解决建设用地不足问题。加强与金融部门的合作，拓宽资金筹措渠道，努力探索金融服务体系建设，支持金融机构加大对中小企业信贷投入，切实缓解企业融资难问题。积极协调和配合供电部门加大电力设施建设力度，争取更多用电指标，逐步缓解我市用电形势偏紧的矛盾。主动为企业搭建平台、提供信息，引导企业加强职工引进、培训力度，改善工作生活环境，构建和谐劳资关系。

（七）加快体制机制创新，努力增强发展活力。继续转变政府职能，深化行政审批制度改革，构建精简高效的政府服务体系。创新政府投资管理方式，完善投资审批、核准和备案制度，实行政府投资项目预算约束制和绩效评价制，推行政府投资项目“代建制”。完善城市资源有偿使用制度，提高公共资源市场化运作水平。推进事业单位和公共事业等领导领域的改革。积极发展行业协会和中介组织，完善公共服务体系。推进市属国有经济布局调整，重组市资产经营（管理）公司和外贸、旅游等企业集团。加强国有资产监管制度建设，完善国有企业业绩考核指标体系。引入战略投资者参与重点骨干国有企业改革，全面完成市属国有中小企业产权改革扫尾工作。

（八）继续抓好市直经济发展，增强市直经济实力。落实加快发展市直经济的扶持激励措施，重点抓好以风华高科、星湖科技、蓝带啤酒、本田金属、亚洲铝业为龙头的电子信息、生物制药、食品饮料、汽车配件、金属加工五大支柱产业，推动市直企业增资扩产、做大做强。加快肇庆高新区建设，发挥省重点工业园区品牌效应，大力引进投资强度大、技术含量高、创税能力强、环保条件好的重大项目，抓好重大项目的促建促产，推进申报国家级高新区和扩园工作。谋划建设市直工业发展新载体，加快鼎湖高新产业集聚基地和肇庆新港临港产业园规划建设，促进市直工业与港口物流业对接互动。加快发展肇庆城区以旅游、房地产、商贸、物流、金融和会展业为重点的第三产业，发展壮大城市经济。

（九）扎实做好财税工作，切实提高发展保障能力。积极培植财源，努力拓展税基，强化税收征管，确保企业在本地依法足额纳税。优化收入结构，降低非税收入比重，提高财政增长质量。调整支出结构，严格控制一般性支出增长，扩大公共财政覆盖面，增加民生投入，切实提高教育、科技、卫生、“三农”、社保等社会公共产品和公共服务保障能力。强化落实部门预算制度、国库集中支付制度、财政支出绩效评价制度。健全政府性债务管理机制。协调帮助企业适应“两税合并”政策调整。

（十）加强资源节约和环境保护，促进可持续发展。强化节能减排责任制，加强环保监测监管，落实节能减排市场准入标准、强制性能效标准和环保标准，建立节能降耗和污染减排的统计、监测、考核办法，确保主要污染物排放量控制在省下达的指标内。强化重点能耗行业和企业的监督，依法强制淘汰落后工艺和产品，推进企业循环经济试点，巩固创模成果。加快污水处理、垃圾无害化处理等环保基础设施建设，做好全国第一次污染源普查，强化环境污染综合整治，突出抓好独立河综合治理，切实保护饮用水源。抓好全国第二次土地调查组织工作。强化土地执法，严查土地违规使用。做好矿产资源开发管理，抓好违规采石场、瓷土场的关闭复绿及水土保持工作。

二、坚持以人为本，重视解决民生问题

（一）深入实施“五大民生工程”，切实为民多办好事实事。推进技能培训工程，抓好全国统筹城乡就业试点。完成下岗再就业培训3000人，农村青年职业技能培训3万人，农民工技能提升培训1.4万人，加强中高级技能人才培养。推进教育均衡发展工程，完善城乡均衡发展的义务教育经费保障机制，建立农村义务教育学校校舍维修长效机制，肇庆城区新建2所小学，增加优质学位3600个。落实城市低保家庭高中学生助学和中职国家助学政策。推进便民廉医工程，提高新型农村合作医疗覆盖面和保障水平，农村常住人口参合率达到99%以上，住院报销封顶线提高到1.5万元以上。加快乡镇卫生院和社区卫生服务机构建设，全面推进村卫生站规范化建设。推进全民安居工程，积极抓好试点，稳步推进农村泥砖房改造。建立健全城镇住房保障制度，端州城区计划安排安居房1000套。推进生产生活环境改造工程，全面启动通500人以上自然村公路“通达工程”。加快农村客运网络建设，争取行政村客运班线通达率达到100%。加快城区“百姓路”、“百姓灯”改造。组织实施18宗农村饮水安全工程。

（二）完善社会保障体系，提高社会保障水平。完善社会养老保险市级统筹，加大社会保险扩面征缴力度。以建立被征地农民养老保险制度为重点，推进农村养老保险制度建设。建立覆盖全体城镇非从业居民的基本医疗保险制度。研究解决早期离开国有集体企业人员社会保险问题。完善城乡居民最低生活保障制度，在应保尽保的基础上提高低保标准。完善五保供养机制，力争五保老人集中供养率提高到30%以上。加强防灾减灾工作，逐步建立完善救灾应急救援体制和庇护场所。加强慈善机构建设，完善慈善长效机制。

（三）关注热点难点问题，解决群众生产生活困难。落实“米袋子”、“菜蓝子”负责制，严格控制出台政府提价项目，加强价格监测，完善价格调节基金征用监管机制，保障市场供给，努力控制物价过快上涨。切实帮助“零就业”家庭成员、“4050”人员、低保人员、残疾人和被征地农民等困难群体解决社会保障、劳动就业、子女就学等问题。认真帮助职工解决住房困难问题。基本完成肇庆城区房产办证难历史遗留问题。加强法律援助和司法救助，强化劳动保障监察执法和劳动争议仲裁工作，引导企业建立工资正常增长和支付保障机制。

三、加快发展社会各项事业，促进社会和谐安康

（一）努力推动科技进步，提高自主创新能力。深入实施人才强市战略，加大人才引进、培养、使用力度。积极构建自主创新体系，重点抓好电子信息、生物工程、汽车配件等高新产业技术创新平台建设。完成市科技中心建设。编制肇庆高新区自主创新中长期发展规划，抓好创业服务中心和以亚铝金属加工为龙头的“国家火炬计划肇庆新材料产业基地”建设。深化产学研合作，组织申报省重点科技项目和粤港关键领域重点突破项目，加快列入广东省与教育部产学研结合项目和示范基地建设。实施科技富民强县工程，提升专业镇技术创新能力。推进果品、林产化工、南药、竹业、水产星火技术产业带建设。推进“农村信息直通车工程”。加强科普、专利工作。

（二）继续优先发展教育，加快建设教育强市。高标准普及九年义务教育，推进规范化学校建设，加快改造城镇薄弱学校，从今年春季学期起全面实行城乡免费义务教育，确保小学和初中适龄人口入学率分别达到99.9%和98.2%以上，适龄残疾儿童少年同步接受义务教育。加快普及高中阶段教育，扩建（新建）一批高中阶段学校，增加高中阶段学位1.52万个，完成普高和中职招生5.98万人。推动高等教育发展，增加特色专业设置，扩大招生规模。办好省级职教基地，支持民办职业教育加快发展，建立和完善中职学生资助体系。重视发展学前教育。全面推进素质教育。

（三）加快建设文化名市，提高社会文明素质。发展文化事业，深入实施全国文化信息资源共享工程、农村电影放映工程、广播电视村村通工程、农家书屋建设工程、乡镇综合文化站试点改革等“五大文化工程”。加强文化市场管理，扶持文化旅游、工艺美术、传媒出版、演艺娱乐、文博会展等文化产业，推动端砚文化村等项目建设，打造端砚文化和宋文化特色品牌。着手规划建设市图书馆，推进“数字图书馆”系统建设。做好全国第三次文物普查和端砚联合申报“文房四宝”为世界文化遗产工作，加强对古村落的保护和利用。开展“文化创作年”系统活动，繁荣群众艺术，培育文艺精品。精心策划开展纪念改革开放30年、“迎奥运、讲文明、树新风”等群众性精神文明活动。

（四）切实加强医疗卫生等工作，全面发展社会各项事业。加快公共卫生体系建设，完善卫生监督网络，全面完成疾控机构建设项目。强化重大传染病防控。加强医疗服务和血液质量管理。加快发展中医药事业。健全人口和计划生育工作机制，推进“两无”（镇街无政策外多孩出生，村居无政策外出生）活动，综合治理出生人口性别比偏高问题，确保完成人口与计划生育工作目标。做好全国第二次经济普查准备工作。支持我市入选奥运优秀健儿备战训练，掀起全民健身活动新高潮。加强国防动员、民兵预备役和人防建设，深入开展省双拥模范城（县）创建活动。切实维护妇女儿童和残疾人合法权益。落实民族和宗教政策。继续做好民政、外事、侨务、法制、统计、档案、老龄等工作。

（五）提高社会管理水平，确保社会和谐稳定。开展平安创建活动，加强社会治安综合治理，着重推进社会治安防控体系尤其是视频监控系统建设，加强流动人口、出租屋的服务和管理，依法打击和严惩各类犯罪分子，提高人民群众的安全感。加强信访和维稳工作，建立重大事项维稳风险评估制度，深入排查调处矛盾纠纷，有效预防和妥善处置群体性事件。加大产品质量和食品安全整治力度，构建产品质量安全长效机制。健全公共安全行政管理机制，强化危险化学品、矿山、交通、建筑施工等重点行业和领域安全整治。完善应急体制和应急预案，提高应对突发事件能力。

四、强化公共服务型政府建设，不断提高行政服务水平

（一）加强民主法制建设，提高依法行政水平。自觉接受人大及其常委会的监督，执行市人大及其常委会的决议，向人大及其常委会报告工作和向人民政协通报情况。办好人大代表议案、建议和政协建设、提案。认真听取民主党派、工商联、无党派人士和人民团体的意见。深入开展“五五”普法宣传教育。巩固扩大基层民主，完成村、社区两委换届选举，建设和谐社区。坚持依法行政，严格依法办事。完善重大决策专家咨询、重大事项社会公示和听证、新闻发布等制度。做好行政复议工作。落实行政执法责任制，加强行政执法监督。

（二）加强政府作风建设，优化行政服务环境。继续解放思想，努力争当实践科学发展观排头兵，营造干事创业、敢于负责、勇于开拓、务实有为的良好氛围。加快转变政府职能，促进依法行政，坚持公正办事。推进政务、村务、厂务公开电子政务建设。抓好“行风评议”，办好“行风热线”。落实年度工作考核目标责任制，加强行政效能监察，精简各类会议、文件，提高机关效能，增强执行力、协调力、服务力和监督力。

（三）加强公务员队伍建设，提高化解矛盾和解决问题能力。贯彻实施《公务员法》和《公务员考核暂行规定》，完善公务员选人用人机制和考核指标体系。加强学习型机关建设，强化公务员学习培训。增强公务员公仆意识，切实纠正滥用权力、办事拖沓、效率低下等行为，进一步提高服务效率，切实为民多办好事实事。规范公务员工资待遇。加强调查研究，及时发现解决经济社会发展和涉及群众切身利益的热点难点问题。

（四）加强廉政建设，树立政府良好形象。落实廉政责任制，认真执行《建立健全教育、制度、监督并重的惩治和预防腐败体系实施办法》，严肃查处违纪违法案件。加强审计监督，规范公务消费，制止铺张浪费。完善农村党风廉政建设信息公开平台。坚决纠正损害群众利益的不正之风。

各位代表！党的十七大为我们指明了前进方向。我们要更加紧密地团结在以胡锦涛同志为总书记的党中央周围，高举中国特色社会主义伟大旗帜，坚持以邓小平理论和“三个代表”重要思想为指导，深入贯彻落实科学发展观，在中共肇庆市委的正确领导下，解放思想，开拓创新，扎实工作，全面完成今年的各项任务，为建设繁荣活力、文明法治、和谐安康、生态环保肇庆而努力奋斗！
